# Issues and recommendations for the residual approach to quantifying cognitive resilience and reserve

**DOI:** 10.1186/s13195-022-01049-w

**Published:** 2022-07-25

**Authors:** Jeremy A. Elman, Jacob W. Vogel, Diana I. Bocancea, Rik Ossenkoppele, Anna C. van Loenhoud, Xin M. Tu, William S. Kremen, the Alzheimer’s Disease Neuroimaging Initiative

**Affiliations:** 1 Department of Psychiatry, University of California San Diego, 9500 Gilman Dr. (MC0738), La Jolla, CA 92093 USA; 2 Center for Behavior Genetics of Aging, University of California San Diego, La Jolla, CA USA; 3 Penn/CHOP Lifespan Brain Institute, University of Pennsylvania, Philadelphia, PA USA; 4 Department of Psychiatry, University of Pennsylvania, Philadelphia, PA 19104 USA; 5 Alzheimer Center Amsterdam, Department of Neurology, Amsterdam Neuroscience, Vrije Universiteit Amsterdam, Amsterdam UMC, Amsterdam, the Netherlands; 6 VU University Medical Center, Amsterdam, the Netherlands; 7 Clinical Memory Research Unit, Lund University, Lund, Sweden; 8 Family Medicine and Public Health, University of California San Diego, La Jolla, CA USA

**Keywords:** Resilience, Residuals, Cognitive reserve, Alzheimer’s disease

## Abstract

**Background:**

Cognitive reserve and resilience are terms used to explain interindividual variability in maintenance of cognitive health in response to adverse factors, such as brain pathology in the context of aging or neurodegenerative disorders. There is substantial interest in identifying tractable substrates of resilience to potentially leverage this phenomenon into intervention strategies. One way of operationalizing cognitive resilience that has gained popularity is the residual method: regressing cognition on an adverse factor and using the residual as a measure of resilience. This method is attractive because it provides a statistical approach that is an intuitive match to the reserve/resilience conceptual framework. However, due to statistical properties of the regression equation, the residual approach has qualities that complicate its interpretation as an index of resilience and make it statistically inappropriate in certain circumstances.

**Methods and results:**

We describe statistical properties of the regression equation to illustrate why the residual is highly correlated with the cognitive score from which it was derived. Using both simulations and real data, we model common applications of the approach by creating a residual score (global cognition residualized for hippocampal volume) in individuals along the AD spectrum. We demonstrate that in most real-life scenarios, the residual measure of cognitive resilience is highly correlated with cognition, and the degree of this correlation depends on the initial relationship between the adverse factor and cognition. Subsequently, any association between this resilience metric and an external variable may actually be driven by cognition, rather than by an operationalized measure of resilience. We then assess several strategies proposed as potential solutions to this problem, such as including both the residual and original cognitive measure in a model. However, we conclude these solutions may be insufficient, and we instead recommend against “pre-regression” strategies altogether in favor of using statistical moderation (e.g., interactions) to quantify resilience.

**Conclusions:**

Caution should be taken in the use and interpretation of the residual-based method of cognitive resilience. Rather than identifying resilient individuals, we encourage building more complete models of cognition to better identify the specific adverse and protective factors that influence cognitive decline.

## Background

Studies of aging and neurodegenerative disease often find that trajectories of cognitive and brain decline are highly heterogeneous. The concepts of resilience and reserve have been used to partially explain variable outcomes with respect to aging and disease [1–6]. Some individuals may start with higher cognitive ability and thus take longer to reach a given threshold of impairment. Alternatively, some individuals may be less vulnerable to the negative effects of aging or disease-related brain changes. Here, we focus on this latter concept which, we refer to as *cognitive resilience* [7]. Thus, an individual that can sustain better cognitive function despite some level of adverse factor is considered to have a high level of cognitive resilience. Understanding the mechanisms that allow such resilience may point to intervention strategies to slow or prevent decline.

One approach that has been increasingly used to operationalize resilience is to calculate the residuals from a regression of cognitive performance on one or more putative measures of brain decline or pathology such as hippocampal volume or β-amyloid [8]. This approach has been of particular interest in the context of Alzheimer’s disease (AD), with a recent meta-analysis finding that higher resilience as indexed by residual measures is associated with reduced risk of dementia or AD [9]. The mapping of conceptual to operational definitions in the study of reserve and resilience is often inconsistent. What makes the “residual approach” appealing is that the statistical interpretation of these residuals very closely matches the conceptual interpretation of resilience: high or low residuals reflect cognitive performance that is higher or lower than expected given the level of adverse factor, respectively. The initial paper by Reed et al. introducing this approach contained a nuanced discussion of how to interpret the residual and whether it should be considered a measure of reserve (or under our definition, resilience). The authors alluded to several potential limitations, one of which we explore here.

If residuals are to be a useful index of resilience, they should tell us something different than our original measure of cognitive performance. That is, whether or not someone is resilient should not depend on their absolute level of cognitive performance, it should only reflect whether they are performing better or worse than expected given an adverse factor. Despite its sound conceptual grounding, the residual approach to operationalizing resilience fails to meet this specific criteria. This issue has been raised previously [10], but given the continued and widespread interest in the residual approach, a more extensive examination of the issue and proposed solutions is warranted. We first demonstrate the source of this non-independence and its magnitude across several scenarios using simulated and real data. We then examine several approaches that have been proposed to correct for this issue taken from the brain age literature. Finally, we discuss potential alternative approaches and future directions for study.

## Methods and results

The following section uses a combination of simulated and real-world data to illustrate issues and considerations for the residual approach. In order to provide a logical progression through these concepts, we intermix descriptions of methods and results organized around each point.

### Non-independence of residuals and cognitive performance

We can examine how the residual is typically calculated using the standard regression formula. Let *y*_i_ denote individual *i*’s score on a cognitive test and *x*_i_ an adverse factor such as level of amyloid or brain atrophy:

$${y}_i={x}_i\beta +{\delta}_{1i}$$

where *β* is the regression coefficient reflecting the strength of association between the adverse factor and cognitive score and *δ*_*1*_ denotes the residual, or error term, for the unexplained variability in *y*_i_ by *x*_i_. We then solve for *δ*_*1*_ to obtain:

$${\delta}_{1i}={y}_i-{x}_i\beta$$

This residual is then used as an index of resilience in subsequent analyses. For example, it may be used as a predictor of progression to dementia, or it may be used as an outcome when the goal is to identify what factors contribute to or are associated with resilience.

Although *δ*_*1*_ is by definition uncorrelated with *x*_*i*_, our adverse factor, it will almost certainly have some correlation with *y*, our measure of cognition. The magnitude of this correlation is dependent on the strength of *β*, the association between cognition and the adverse factor. When the association between these two variables is small (and thus *β* approaches 0), the residual measure *δ*_*1*_ will be more highly correlated with our cognitive measure *y.* In the extreme case where *y* and *x* are completely unrelated, *β* will be 0 and the resulting residual score will simply be *y*, our cognitive score (or a mean-centered version of *y*, assuming intercept is included in the regression). At the other extreme, where *x* perfectly predicts *y*, the variance of our residual will shrink to 0, at which point it no longer provides any information whatsoever. However, error in our *x* measure will shrink *β* towards 0, known as *regression dilution*, or attenuation [11, 12]. Thus, we are more likely to encounter the former scenario than the latter.

**Fig. 1:**
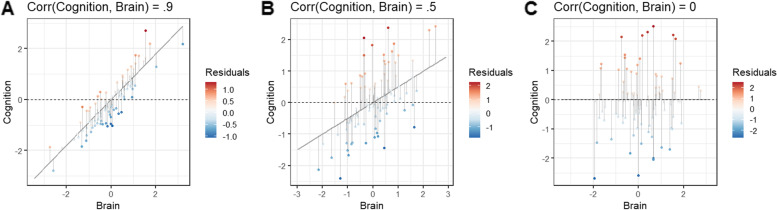
Examples of residuals from two extreme scenarios. One thousand paired values were generated from a multivariate normal distribution to represent a cognitive score and brain measure indicative of atrophy. Variables in panels **A** and **C** were generated with correlations of *r* = 0.9 and *r* = 0.0 to show extreme scenarios. The variables in panel **B** were generated with a correlation of *r* = 0.5 to reflect what would be considered a strong yet realistic association between brain and cognitive measures. The cognitive score was regressed on the brain measure, and residuals from 100 randomly selected observations (for easier visualization) are plotted with color indicating the magnitude of the residuals (i.e., distance between predicted cognitive score and actual cognitive score). The dashed line represented the average cognitive score. When cognition and the brain are highly correlated (panel **A**), individuals with both higher and lower than average cognitive scores display a mix of positive and negative residuals. In contrast, when cognition and the brain are uncorrelated, individuals with high cognitive scores all have positive residuals and individuals with low cognitive scores have negative residuals

These concepts are illustrated in Fig. 1 using simulated data. We generated 1000 pairs of *y*_i_ and *x*_i_ from a bivariate normal distribution, with low, medium, or strong correlations between the variables. We will consider these to represent a cognitive measure (*y*) and a brain measure (*x*) corresponding to some adverse factor such as hippocampal atrophy. The individual data points are colored red or blue for positive or negative residuals, with the intensity corresponding to how large the residual value is (i.e., deviation from the expected value along the regression line). In Fig. 1A, the cognitive and brain measures are correlated at *r* = 0.9. The resulting scenario is what we intuitively expect in that individuals with higher or lower than average cognitive performance (above or below the dashed line) show a mix of positive and negative residuals of varying magnitude. Figure 1B shows what would be considered a relatively strong association between cognitive and brain variables in real-world scenarios (*r* = .5). Here, there is still some mixture, but individuals with higher cognition tend to also have higher residuals. Figure 1C shows a scenario in which the cognitive and brain measures are completely uncorrelated (*r* = 0). In this case, individuals with higher-than-average cognition all have positive residuals. It becomes clear that the magnitude of the residual is simply the deviation of an individual’s score from the mean cognitive score.

**Fig. 2:**
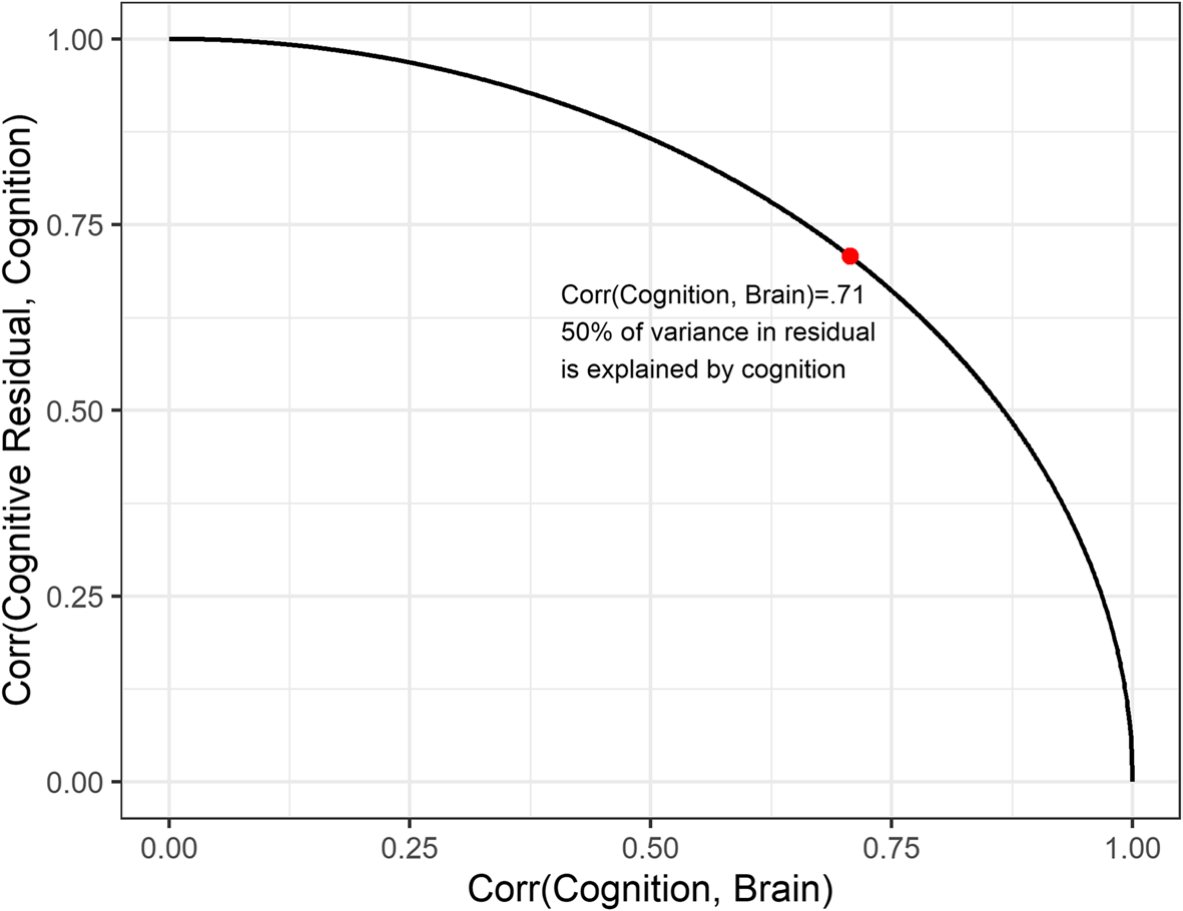
Correlation between cognition residual and cognition varies as a function of correlation between cognition and an adverse factor. One thousand paired values representing a cognitive score and brain measure indicative of atrophy were generated from a multivariate normal distribution with a correlation varying from 0 to 1. At each iteration, cognition was regressed on the brain measure and residuals were saved. The plot displays the correlation of residuals with the cognitive score and the association between cognition and brain increases. The red dot marks the point where the correlation between cognition and brain is 0.71. When the correlation between cognition and brain is smaller than this value, cognition will explain >50% of the variance in the residual measure (i.e., squared correlation between the two variables)

We can examine how the correlation between the residual (*δ*_*1*_) and the original cognitive score (*y*) varies as the correlation between cognitive score (*x*) and brain measure (*y*) varies. As before, we simulated sets of 1000 paired scores from a bivariate normal distribution with the correlation varying from 0 to 1. For each specified correlation, we create a residual score as described above and then calculate the correlation between this residual (*δ*_*1*_) and the original cognitive score (*y*). Figure 2 shows how Corr(*δ*_*1*_*, y*) varies as a function of Corr(*x, y*). We can see that when the correlation between cognition and the brain is ~0.7, the correlation of cognition and the residual is also ~0.7. In other words, when the correlation between cognition and the brain is less than *r* = 0.7, as is almost always the case, cognition will explain greater than 50% of the variance in the residual (i.e., squared correlation between the two variables).

The above point is further demonstrated using real data from the Alzheimer’s Disease Neuroimaging Initiative (ADNI; http://adni.loni.usc.edu/). We selected 839 individuals with a baseline diagnosis of cognitively normal (*n* = 175), mild cognitive impairment (*n* = 437), or AD dementia (*n* = 227) with evidence of amyloid-β (Aβ) neuropathology (determined based on 11C–Pittsburgh compound B or 18F-florbetapir PET if available, or CSF Aβ42 otherwise), structural MRI and baseline neuropsychological assessment within 6 months from the MRI scan (total sample average age 73.9 ± 7.2 years, 46% females, median education level 16 [range 14–18] years). We selected the baseline ADNI-MEM composite memory score [13] and hippocampal volume as our cognitive and brain variables of interest, the latter measured as SPM12 segmented gray matter volume in a bilateral hippocampal mask based on the AAL atlas and adjusted for total intracranial volume. We select these specific variables so as to simulate a common framework for real-world studies analyzing resilience in the context of AD. We note here (and discuss further below) that it is commonplace in such studies to represent adverse factors as a single variable, and we do so here. However, this may represent a limitation itself, as one variable can rarely represent the totality of pathological insult, meaning some of the residual could be explained by other brain-related pathological insults. All procedures were approved by the Institutional Review Board of participating institutions and informed consent was obtained from all participants.

**Fig. 3:**
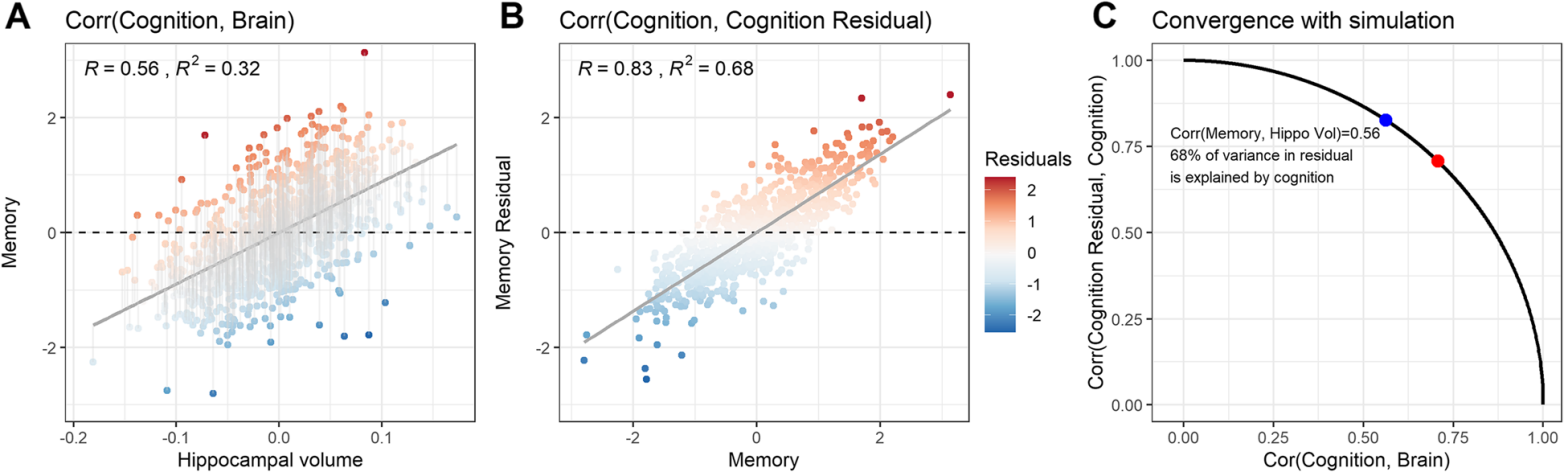
Examples of residuals from the ADNI dataset. Cognition residuals were calculated in a sample of Aβ+ individuals on the Alzheimer’s disease spectrum from the ADNI dataset. **A** ADNI-MEM, a composite measure of memory ability, was regressed on hippocampal volumes derived from structural MRI. Residuals are plotted with color indicating their magnitude. This figure represents a realistic association between the brain and cognition that can be expected in real samples. Panel **B** illustrates the high dependency of the cognition residual on cognition itself, with their correlation being 0.83 in the ADNI example. The blue dot in panel **C** represents the correlation between ADNI-MEM and hippocampal volume, for which 68% of the variance in the resulting residual is explained by the cognition variable. The red dot marks the point where the correlation between cognition and brain is 0.71 and therefore where 50% of the variance in the residual would be explained by cognition (i.e., squared correlation between the two variables). The blue dot falls perfectly along the simulated curve

Baseline ADNI-MEM (cognitive score; *y*) was regressed on hippocampal volume (adverse factor; *x*), and no other (e.g., demographic) covariates were included in the calculation of the residuals (*δ*_*1*_). Despite a relatively strong association between ADNI-MEM and hippocampal volume (*r* = 0.56; Fig. 3A), the model residual (our “resilience measure”) retained a very strong correlation with ADNI-MEM (*r* = 0.83; Fig. 3B), falling precisely along the simulated curve from Fig. 2 (Fig. 3C). Therefore, we cannot determine if better memory than expected given one’s hippocampal volume predicts much of anything that would not already be predicted by simply looking at memory alone.

Unfortunately, our measures of cognition and adverse factor rarely approach correlations of *r* = 0.7. This means the majority (often most) of the variance in residual-based resilience measures is usually shared with cognition. While this is not strictly problematic, it may not be appropriate in many scenarios, for example when a secondary variable of interest is correlated with or dependent on cognition. In such a circumstance, we cannot differentiate whether the association with the external variable is driven by a pre-existing association with cognition or a unique resilience factor without further modeling these terms. Longitudinal cognitive decline or clinical diagnosis (when diagnosis is partially or entirely based on cognitive scores) are two frequent examples of this type of external variable. In such a scenario, a model may reveal a strong relationship between “resilience” and cognitive decline that may actually be driven by the correlation between baseline cognition and cognitive decline.

**Fig. 4:**
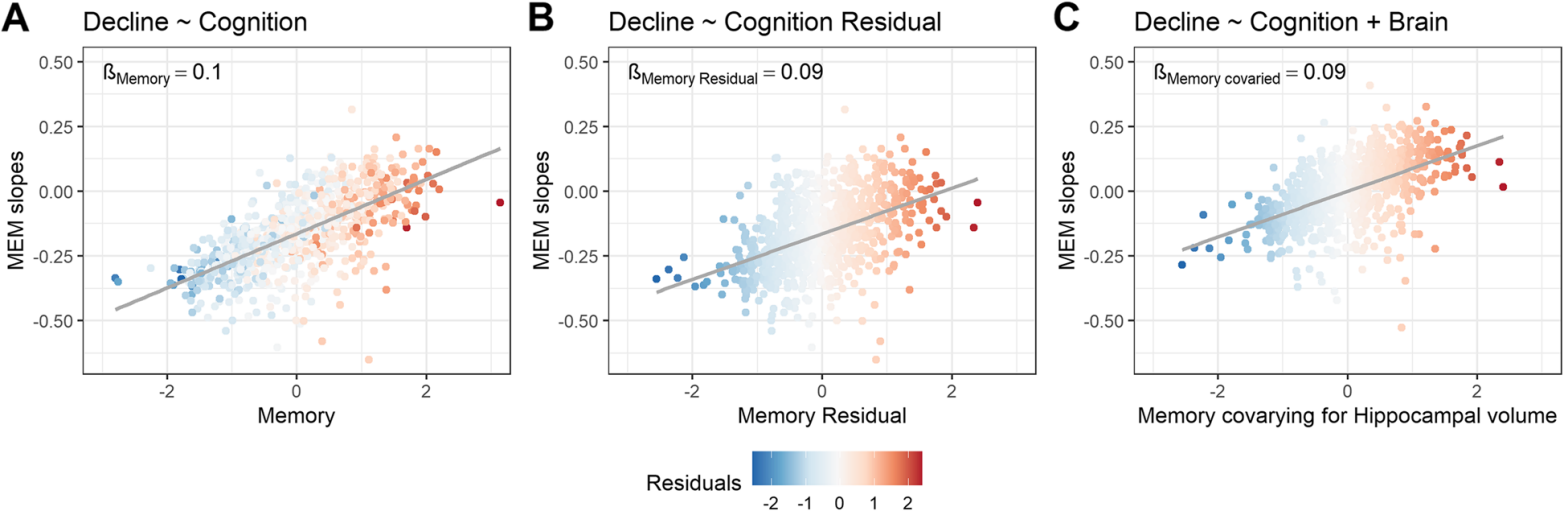
Associations with cognitive decline. Figures represent associations between annual slopes in memory (derived as the random slopes from a linear mixed effects model of memory regressed on time that included the whole sample) and **A** memory at baseline, **B** residuals of baseline memory regressed on hippocampal volume, and **C** baseline memory when hippocampal is also included as a predictor in the model. This figure illustrates that the regression coefficient (*β*) estimated for the memory residual in panel **B** (a linear regression of the form “Slopes ~ Residual”) is equivalent to the coefficient of the cognition term in a multivariable regression that includes both cognition and brain as predictors of decline (slopes ~ memory + hippocampal volume) shown in panel **C**. Note that panel **C** is a partial regression plot, in which the data points illustrate the relationship between memory slopes and cognition when covarying for brain. MEM = ADNI memory factor score

In another example demonstrating this point in ADNI data, we compare results from three models, each using the following predictors: baseline memory only (Fig. 4A), the residual score only (hippocampal volume regressed out of memory performance prior to entry into the model; Fig. 4B), and baseline memory and hippocampal volume both used as predictors in the same model (Fig. 4C). We show that baseline cognitive performance (i.e., memory in this example) shows a high correlation with longitudinal memory change (Fig 4A), the latter estimated using linear mixed effect models with random intercepts and slopes per participant. As an illustration of the above point, we find a strong relationship between memory change and the pre-regressed memory residual (Fig. 4B). One can appreciate the difficulty in determining the degree to which this strong relationship is driven by a pre-existing strong relationship between memory decline and the original memory measure from which the residual was derived (Fig. 4A), because these two predictors are highly collinear.

When modeling cognition (memory score; previously denoted *y*) and brain (hippocampal volume; previously denoted *x*) measures together in a multivariable regression of memory decline (Fig. 4C), the resulting term for the memory score in this situation is statistically equivalent to using the pre-regressed residual (*δ*_*1*_). Accordingly, the regression coefficients (*β*) for the two modeling approaches illustrated in Fig. 4B and C are identical. Importantly, this highlights the fact that one is essentially regressing longitudinal cognitive decline onto baseline cognition (controlling for hippocampal volume) rather than some unique entity. Extending the residual-based resilience measure to external variables with unknown relationship to *y* can also lead to ambiguous interpretations. Given the high collinearity with cognition, one would not be able to easily disambiguate whether resulting associations were specific to resilience or simply cognition-related. Unfortunately, as will be described below, approaches to correct for this, such as adding *y* as a covariate into the model, may not surmount the issues described here.

### Similarities with the brain age literature

The issue described above has been discussed extensively in the brain age literature. These studies attempt to predict chronological age using a combination of brain features measured with MRI. The trained model is then used to predict age in a new sample with the same brain features. The deviation from predicted age is known as the brain age gap or predicted brain age difference and has been proposed to reflect accelerated or decelerated brain aging. However, it is well-known that this brain age gap is correlated with chronological age [14–18]. For example, younger individuals will tend to have a predicted brain age older than their chronological age and therefore an advanced brain age gap. The opposite is true for older adults. This occurs for statistically identical reasons as seen in the residual approach to cognitive resilience. If we substitute chronological age (*y*), brain features (*x*_*1,*_ *x*_*2*_ *…x*_*n*_), and brain age gap (*δ*_*1*_) into our previous formulas, we can see that this correlation becomes stronger when brain features do not strongly predict chronological age.

### Calculating an independent residual

Solutions to this problem are primarily discussed in the context of brain age, where it has been considered in detail [14–16, 18–21]. We refer readers to these papers for more details, but there are two primary approaches proposed to correct or adjust for correlation with our *y* variables. The first is that we can simply include *y* as a covariate when *δ*_*1*_ is used as a predictor. We can then consider the effect of our residual to be independent of our original measure (e.g., resilience independent of cognitive performance level). However, these variables are likely to be collinear; in the extreme case where *y* and *x* are unrelated, the predictors will be perfectly collinear. Collinearity between predictors can be problematic, resulting in unstable or imprecise estimates. It can also result in sign flipping [22]. We can understand this sign flipping from a conceptual point of view by considering what happens when we include our original cognitive measure with the resilience measure in the model. The coefficient of the resilience measure would be interpreted as the effect when individuals are equated on cognitive performance. If individuals are equated on cognitive performance, then any variance in their resilience measure must be driven by variance on the adverse brain factor. Therefore, a higher resilience score in this model would simply reflect higher levels of the adverse factor (e.g., atrophy), and the effect of such a score interpreted in this way may be expected to have the opposite effect of our resilience score unadjusted for cognitive performance.

We once again demonstrate this phenomenon with the ADNI data described above (Table 1). Several models were run to predict the decline on the ADNI-MEM score. When the residual score is the only predictor, there is a positive association such that higher residuals (i.e., greater resilience) predict less decline. However, when the original cognitive measure is included in the measure, the sign of the coefficient for the residuals becomes negative because higher values now reflect more hippocampal atrophy. Additionally, this approach does not produce a single residual measure, which many studies seek to use in subsequent analyses as an outcome variable or entry as one feature in a multivariate model.

**Table 1:** Relationship of residuals with cognitive decline, with and without adjusting for baseline cognition

	β (residual*time)	***CI***	***p-value***
Model 1: Cognition ~ Residuals*Time	0.05	0.03–0.08	**<0.001**
Model 2: Cognition ~ Residuals*Time + BaselineCognition*Time	−0.10	−0.14 to −0.06	**<0.001**
Model 3: Cognition ~ ResidualsCor*Time	−0.11	−0.14 to −0.07	**<0.001**

All models are linear mixed effects models with random intercepts and slopes per participant, and cognitive score at each timepoint as dependent variable. Cognition was assessed using ADNI-MEM, the standardized composite ADNI memory score

Model 1: Cognition ~ Residuals*Time. This model represents the usual case scenario in which the (cognition) residual is used as a predictor in a subsequent analysis (e.g., here to predict decline)

Model 2: Cognition ~ Residuals*Time + BaselineCognition*Time. This model represents the first proposed alternative solution, i.e., adding baseline cognition, on which the residual is based, as a covariate alongside the residual to assess the effect of “resilience independent of cognitive performance”

Model 3: Cognition ~ ResidualsCor*Time. This model represents the second proposed alternative solution, i.e., regressing the cognition variable out of the residual to obtain a “corrected” residual (“ResidualsCor”) in a subsequent analysis

*Abbreviations*: *β* unstandardized regression coefficient from linear mixed effects models, *CI* 95% confidence interval

An alternative approach that does produce a single adjusted score entails regressing the *y* variable out of the residual:

$${\delta}_{1i}={y}_i\gamma +{\delta}_{2i}$$

Solving for *δ*_*2*_ yields our adjusted residual score:

$${\delta}_{2i}={\delta}_{1i}-{y}_i\gamma$$

This new adjusted residual may be considered an index of resilience that is uncorrelated with cognitive performance. Although this appears to match our conceptual definition of resilience, we can explore further by replacing *y*_*i*_ with *y*_*i*_ *= x*_*i*_*β + δ*_*1i*_:

$${\delta}_{2i}={\delta}_{1i}-{y}_i\gamma ={\delta}_{1i}-\left({x}_i\beta +{\delta}_{1i}\right)\gamma ={\delta}_{1i}\left(1-\gamma \right)-{x}_i\beta \gamma$$

Thus, our adjusted residual *δ*_*2i*_ contains our brain measure *x*_*i*_, resulting in a negative correlation between the two. The magnitude of this correlation will be proportional to Corr(*δ*_*1*_*, y*), albeit with the reverse sign. This flip in sign occurs for the same reasons as described above. In other words, we shift from a measure of resilience that is correlated with our cognitive score to one that is correlated with our adverse factor. This is likely not the desired measure from a conceptual standpoint.

## Discussion

### Alternative solutions

We have shown here that, in most real-word cases, residual-based methods of measuring resilience are highly collinear with the dependent variable (i.e., cognition). This means that the residual measure is rarely representative of resilience and can cause issues with interpretations depending on how it is used in subsequent analyses. As an alternative, one may avoid calculating the residual at all and instead examine how a third variable moderates the association of an adverse factor with cognition. Testing for an interaction effect has previously been a recommended approach to examining cognitive resilience [1, 2]. The interaction effect will capture the degree to which this third variable systematically explains deviation in individuals’ cognitive score relative to what is expected given some adverse factor. We may then interpret the interaction as evidence of cognitive resilience and the moderator as a factor contributing to resilience. Importantly, this model includes both the interaction and main effects of each variable involved. Creating a residual of cognitive decline (i.e., an estimate of how an individual’s slope differs from the group mean) faces the same issues we describe in the cross-sectional case. However, the interaction approach can be extended to longitudinal designs by including a three-way interaction that tests the degree to which a resilience factor minimizes the impact of an adverse factor on cognitive decline. Two-step approaches that pre-regress out covariates not only are less parsimonious but can also lead to confusion in interpretations, can improperly represent degrees of freedom, and may not be necessary at all in well-powered studies.

The use of the interaction approach comes with several caveats. First, the distributional properties of the included measures should be assessed. Measures with strong ceiling or floor effects may not be appropriate. For example, if individuals are performing at ceiling, it will not be possible to detect performance that is better than expected. Second, the interaction approach, like the model including both the residual and cognitive score, does not provide a standalone measure of resilience for each individual that can be further investigated for translation to the clinic. This may not necessarily be a drawback — while it is certainly of interest to understand which individuals are exhibiting resilience, we ultimately want to understand what factors contribute to or confer this resilience. Using the interaction approach, we are limited to identifying resilience at the group level (i.e., the interaction effect), but we retain the ability to quantify factors that may drive this resilience at the individual level (i.e., subject-specific values on the moderator variable). It is these factors that contribute to overall resilience by mitigating the impact of pathology on cognition that may represent suitable mechanisms to target for interventional strategies.

### Future directions

Re-examining what the cognition residual represents statistically may help reveal a new path forward for quantifying resilience. The residual represents the totality of unexplained variance in the cognitive variable after accounting for an adverse factor. In other words, it is a negative definition. It is important to note that the initial paper by Reed et al. [8] concludes that the residual may be useful but should ultimately be dispensed with in favor of more complete models of cognition. We come to the same conclusion. Rather than isolating this error term and repurposing it as resilience, it may be more fruitful to focus on constructing a more complete model of cognition by maximizing measurement of other adverse and protective factors, directly or indirectly. This may include modeling previous or premorbid cognitive ability, which helps determine whether current cognitive performance represents long-standing individual differences in performance or is the result of decline — something that the residual score cannot assess. Then, putative resilience factors can be iteratively added to the existing cognitive model to see whether they contribute meaningful, independent information. The covariance or interactions of the putative resilience factor with other aspects of the cognitive model can also be considered. This is in some ways akin to the study of normative aging. Aging effects can be seen as phenomena correlated with age that are driven by factors we have not specifically measured or identified. Recent studies conducting comprehensive neuropathologic exams have been able to attribute a substantial portion of late-life cognitive decline to pathology that would otherwise have been labeled normative aging [23, 24]. The study of resilience may be furthered by including protective factors in such models.

**Fig. 5:**
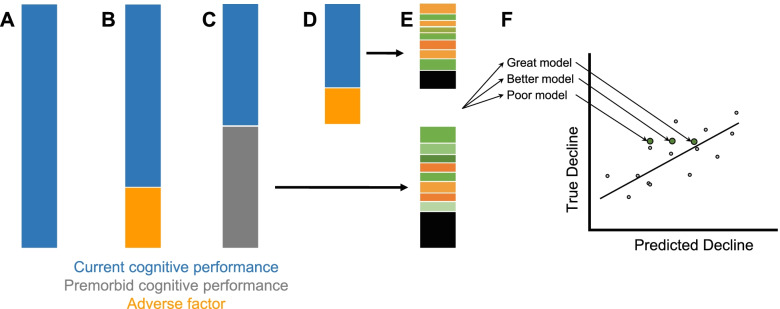
**A** Variance in current cognitive performance (leftmost bar) is driven by a number of contributing factors. **B** If the variance explained by an adverse factor (e.g., hippocampal atrophy, pathology, etc.) is regressed out, the remaining variance is largely the same as the current cognitive performance. **C** A large portion of current cognitive performance is explained by premorbid cognitive performance. The remaining variance can be interpreted as “change in cognitive performance” compared to expected. **D** Variance explained by an adverse factor can be regressed out of this “change in cognitive performance,” but what remains is highly correlated with the original “change in cognitive performance” measure. **E** Variance that remains in current and past cognitive performance can be explained by a host of known and to-be-discovered genetic, environmental, and lifestyle factors and pathologies, as well as measurement noise. Ultimately, our goal is to understand what contributes to this variance and reduce error in our model of cognition. **F** These models can be used to predict cognitive state or forecast cognitive decline. The more comprehensive our models of cognition, the better our individual levels of prediction will be. With better models for cognition, we shift our focus to simulating how modification of a pathological or resilience factor might influence maintenance of healthy cognition

In this way, we encourage those interested in studying resilience to consider reconceptualizing the objectives of these analyses. Modeling the contribution of various adverse and protective factors will allow us to make better predictions about cognitive decline. By continuing to discover and quantify such factors, we can slowly reduce the unexplained variance in cognitive decline (i.e., the residual) and come up with more accurate forecasts of cognitive decline (Fig. 5). In doing so, we can shift our focus from identifying resilient individuals to identifying factors that contribute to better cognitive health. We wish to discover what resilience factors our model predicts will enhance cognitive outcomes if introduced or modified. However, we may also be able to identify factors that improve the early development of cognitive ability or prevent factors over the lifespan that may drive decline in the first place. This pursuit will be enhanced as our model for cognition improves.

Although we recommend against using residual approaches to quantify resilience, we note that these approaches can be appropriate in other contexts such as adjusting a measure for confounding factors. For example, hippocampal volume is often adjusted for individual differences in head size by regressing out intracranial volume, and time to complete Part B of the Trail Making Test may have time to complete Part A regressed out to control for differences in visual scanning and speed. Similarly, regression-based change scores have been used as an alternative to difference scores that account for expected regression to the mean upon re-testing. The critical difference is that these residuals are not interpreted as being independent of the original variable. Rather, they are considered adjusted versions or highly dependent on the original measure.

## Conclusions

The residual approach to measuring resilience has many attractive qualities. However, as seen in the brain age literature, residual measures come with important statistical considerations. As we have shown, these issues complicate interpretability and seriously limit the usefulness of resilience measures in the context of studying cognitive or brain resilience. Although several correction methods have been proposed, these do not appear to produce measures that sufficiently reflect our conceptual idea of resilience as a unique entity. However, understanding the factors that influence resilience is an important goal that will aid in efforts to extend cognitive and brain health spans. Thus, further development of operational definitions of resilience remains a key component to facilitating this work.

## Data Availability

Data used in preparation of this article were obtained from the Alzheimer’s Disease Neuroimaging Initiative (ADNI) database (adni.loni.usc.edu). All data are available upon application and completion of the Data Use Agreement with the ADNI. The datasets used and/or analyzed during the current study are available from the corresponding author on reasonable request.

## Abbreviations

Aβ: Amyloid-β
AD: Alzheimer’s disease
ADNI: Alzheimer’s Disease Neuroimaging Initiative
ADNI-MEM: ADNI composite score for memory
CSF: Cerebrospinal fluid
MRI: Magnetic resonance imaging
PET: Positron emission tomography
